# Deep learning model for differentiating acute and chronic osteoporotic vertebral compressive fractures on multidetector CT: a retrospective study with external validation

**DOI:** 10.3389/fmed.2026.1883238

**Published:** 2026-08-17

**Authors:** Meipeng Min, Xincheng Wei, Kaixiang Yang

**Affiliations:** 1 The Second Affiliated Hospital of Nanjing Medical University, Nanjing, China; 2 The Second Affiliated Hospital of Nanjing University of Chinese Medicine, Nanjing, China

**Keywords:** deep learning, diagnostic rate, image segmentation, multidetector computed tomography, osteoporotic vertebral compressive fractures

## Abstract

**Background:**

Osteoporotic vertebral compression fractures (OVCFs) are a common spinal disease. Differentiating acute and chronic fractures is the key to determining the treatment plan. To develop and evaluate a deep learning model capable of differentiating acute and chronic OVCFs on CT images.

**Methods:**

The internal dataset comprised CT images of 624 fractured vertebrae from 400 patients with OVCF treated at Hospital 1 between January 1, 2020, and November 1, 2023. The patients were randomly divided into a training set (340 patients, 532 fractured vertebrae, 85.0%) and an internal testing set (60 patients, 92 fractured vertebrae, 15.0%). An external testing set included CT images of 86 fractured vertebrae from 70 patients with OVCF treated at Hospital 2 from January 1, 2023, to November 1, 2023, using identical inclusion criteria. Three radiologists manually delineated regions of interest (ROIs) in CT images of diseased vertebrae using Anaconda Prompt software with rectangular bounding boxes. The trained YOLO v7 model’s performance was evaluated on both the internal and external testing sets and compared with that of the three radiologists using metrics with accuracy, sensitivity, specificity, and AUC.

**Results:**

The internal testing set revealed the following: AUC, 0.937 (95%CI: 0.932–0.991); accuracy, 0.961 (0.930–0.977); sensitivity, 0.973 (95%CI: 0.958–0.994); specificity, 0.761 (95%CI: 0.728–0.784); precision, 0.936 (95%CI: 0.920–0.966), and F1 score; 0.954 (95%CI: 0.913–0.975). The external testing set revealed the following: AUC, 0.882 (95%CI: 0.839–0.894); accuracy, 0.852 (95%CI: 0.827–0.886); sensitivity, 0.895 (95%CI: 0.840–0.913); specificity, 0.780 (95%CI: 0.730–0.812); precision, 0.874 (95%CI: 0.839–0.892); and F1 score, 0.884 (95%CI: 0.846–0.903).

**Conclusion:**

The YOLO v7 model achieved good performance for CT-based differentiation of acute versus chronic OVCFs in patients and showed better performance compared to radiologists in both testing sets, which may serve as a decision-support tool for CT-equivocal cases.

## 1 Background

Osteoporotic vertebral compression fractures (OVCFs) represent the most prevalent manifestation of osteoporotic fractures globally, resulting in substantial socioeconomic burdens and significantly diminishing health-related quality of life (HRQoL) and quality-adjusted life years (QALYs) (1–5). The clinical management of OVCFs is inherently complex, requiring tailored therapeutic strategies that account for fracture chronicity, patient comorbidities, age, and treatment goals (6–8). Conservative approaches, including analgesia, bracing, and restricted mobility, remain first-line interventions but are associated with the risks of prolonged immobilization complications such as pneumonia, thromboembolism, and accelerated bone loss (9). Conversely, early surgical intervention in eligible patients – particularly vertebroplasty or kyphoplasty – has demonstrated efficacy in restoring functional mobility and mitigating long-term disability (7). Consequently, precise differentiation between acute and chronic OVCFs is critical for optimizing therapeutic decision-making (10).

Diagnostic challenges persist due to the frequent absence of identifiable trauma and nonspecific symptomatology, often leading to misdiagnosis of OVCFs as degenerative spinal disorders or musculoskeletal strain (11). Radiological evaluation thus serves as the cornerstone for OVCF characterization. MRI scanning is the most suitable diagnostic tool for differentiating acute and chronic OVCFs (12). Fresh OVCFs demonstrate hypointense signal within the affected vertebra on T1-weighted imaging and hyperintense signal on T2-weighted imaging. The presence of bone marrow oedema, which is visualized as hyperintensity in fat saturation sequences (STIR, DIXON), serves as an imaging biomarker for acute or unhealed chronic fractures (9, 13). However, MRI utilization is constrained by cost, contraindications (e.g., metallic implants), and limited accessibility in resource-constrained settings (9). While digital radiography (DR) provides rapid morphological assessment of vertebral collapse, its insensitivity to marrow edema limits its utility in distinguishing acute from chronic fractures (14). High sensitivity to calcified and ossified tissue, and low economic costs make CT an excellent modality for the diagnosis of vertebral fractures (15). Although CT enhances trabecular architecture visualization and detects fracture-related features (e.g., cortical step-offs and retro-pulsed fragments), a substantial subset of acute OVCFs presents with bone marrow edema (BME) without overt cortical disruption, which called bone contusion or incomplete fracture. In these cases, CT may appear normal or show only subtle, non-specific trabecular density changes, leading to diagnostic uncertainty (16). Consequently, radiologists and orthopedic surgeons, especially those with less experience, express a lack of confidence in evaluating acute OVCF based on CT imaging results (17).

Radiomics – a computational paradigm extracting high-dimensional quantitative features from medical images – has shown preliminary promise in fracture characterization (18, 19). However, traditional radiomic workflows for vertebral fracture analysis exhibit the following critical limitations: (1) region-of-interest (ROI) placement often excludes biomechanically critical trabecular microarchitecture due to reliance on sagittal midline sampling (20); and (2) reported model accuracies lack robustness, compounded by insufficient external validation (21). Deep learning (DL) methodologies, especially convolutional neural networks (CNNs), offer transformative potential by automating hierarchical feature extraction directly from raw imaging data. Despite advances in DR-based fracture detection (22), DL applications for CT-based acute OVCF differentiation remain underexplored, with existing models limited by single-center datasets and inadequate generalizability.

To address these gaps, we developed a YOLO v7 model optimized for automated discrimination of acute versus chronic OVCFs using CT imaging. Our model incorporates axial multi-slice CT scanning to capture spatially resolved trabecular injury patterns, overcoming the sampling biases of conventional radiomics. Furthermore, we rigorously validated the model’s performance across heterogeneous external datasets to assess its clinical translatability.

## 2 Materials and methods

### Study design and data collection

**FIGURE 1:**
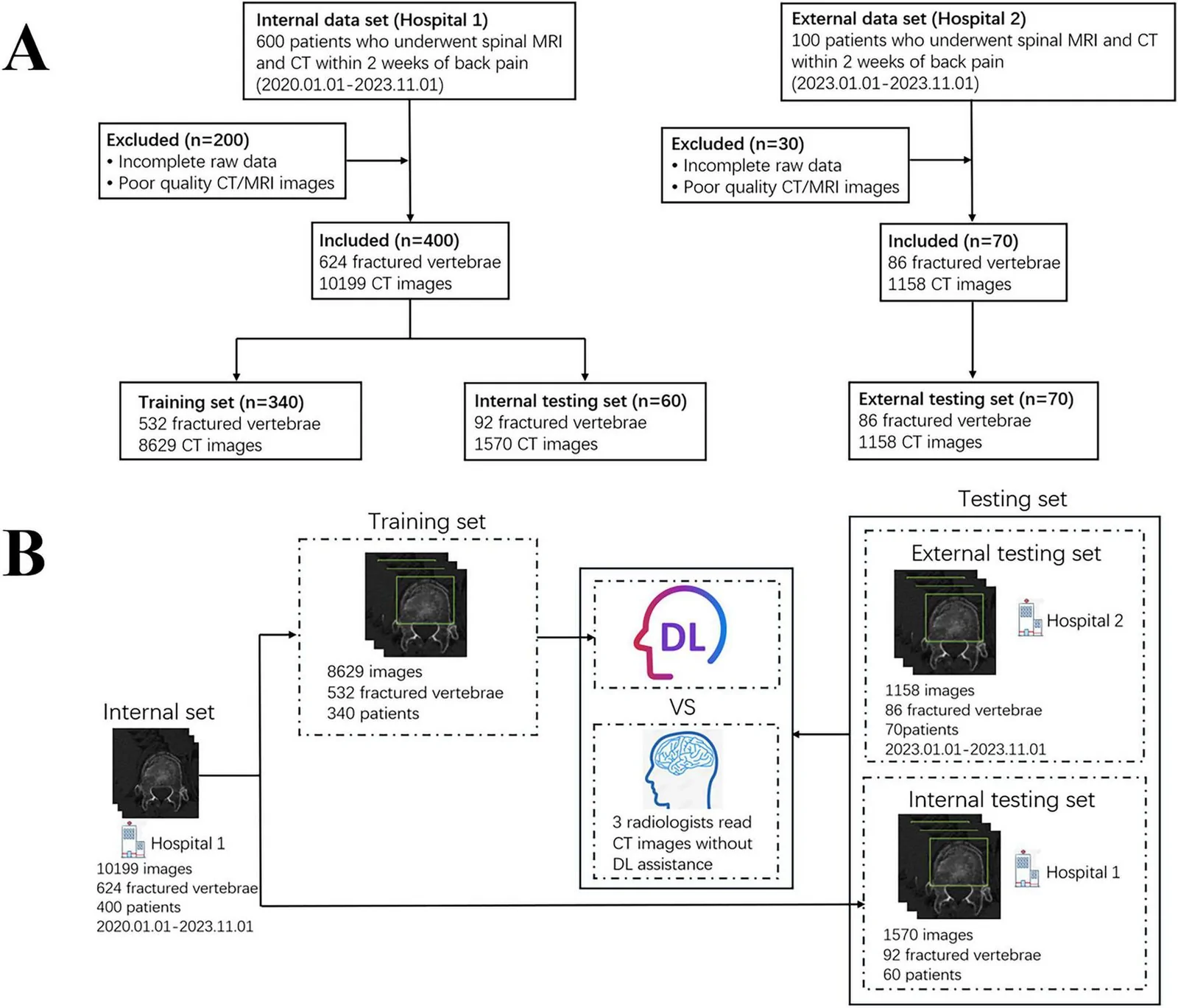
Flowchart of the study design. **(A)** The internal data set was derived from hospital 1, and included a total of 10,199 images of 624 fractured vertebrae from 400 patients selected according to the inclusion and exclusion criteria. The patients were randomly divided into a training set and an internal testing set in a ratio of 85%:15%. The external data set was derived from hospital 2, with a total of 1158 images of 86 fractured vertebrae from 70 patients selected according to the inclusion criteria and exclusion criteria. **(B)** The internal data set was divided into a training set and an internal test set, which are used to develop and train the deep learning model. Use the external testing set to check the performance of the deep learning model. Three radiologists read images from the internal testing set and the external testing set without deep learning assistance, and their performance was compared with that of the deep learning model.

Participants from two hospitals were retrospectively included in this study (Figure 1A). The inclusion criteria were participants who underwent both MRI and CT spine examinations within 1 week (mean time, 5 ± 1.2 days [SD]). The exclusion criteria were participants who did not have complete original data and qualified imaging data. Consequently, the model’s performance is validated exclusively in an MRI-eligible subpopulation, which does not fully represent the target population in which the model would ideally be deployed (i.e., MRI-ineligible or MRI-unavailable settings). To improve model generalizability, our inclusion criteria permitted chronic, suspected malignant, and previously operated (e.g., vertebroplasty, fusion) OVCF cases. Finally, data of 400 patients treated in Hospital 1 from November 2020 to July 2022 were included. 85% of the cases (340 patients, 532 fractured vertebrae and 8629 CT images) were randomly selected for training, while the remaining 15% (60 patients, 92 fractured vertebrae and 1570 CT images) were used as an internal test set to evaluate the performance of the model. For external validation, we further evaluated the model’s performance on an external dataset (70 patients, 86 fractured vertebrae and 1158 CT images) from Hospital 2. The performance of the model was compared with that of three radiologists (Figure 1B). In this study, more CT images of acute OVCFs were selected than those of chronic OVCFs. We considered that in clinical practice, there were more cases of acute OVCF than chronic OVCF, which is in line with the proportion standard of the real clinical environment.

### Image labeling and quality control

We retrieved CT scans of the thoracic or lumbar spine from the hospital’s Picture Archiving and Communication System (PACS) for export in DICOM format. To develop and evaluate the model for lesion segmentation, three physicians with over 10 years of experience performed manual segmentation of lesion areas on the CT images in the dataset at the pixel level. All axial CT images from the fractured vertebra were captured using a DICOM Viewer software and saved in PNG format. This includes slices from patients with acute OVCF and slices from patients with chronic OVCF. All CT images underwent a hierarchical annotation system consisting of three physicians with over 10 years of experience, who validated and corrected the image labels. The annotations were generated using Anaconda3 (version 2022.05) and saved in txt format.

MRI results were used as a reference standard to differentiate between acute and chronic OVCFs. Acute vertebral fractures were defined clinically by acute back pain (<4 weeks) with MRI confirmation of bone marrow edema (BME). BME was radiologically characterized by T1 hypointensity, heterogeneous T2 signal, and hyperintensity on T2 fat-suppressed sequences.

### DL model development

**FIGURE 2:**
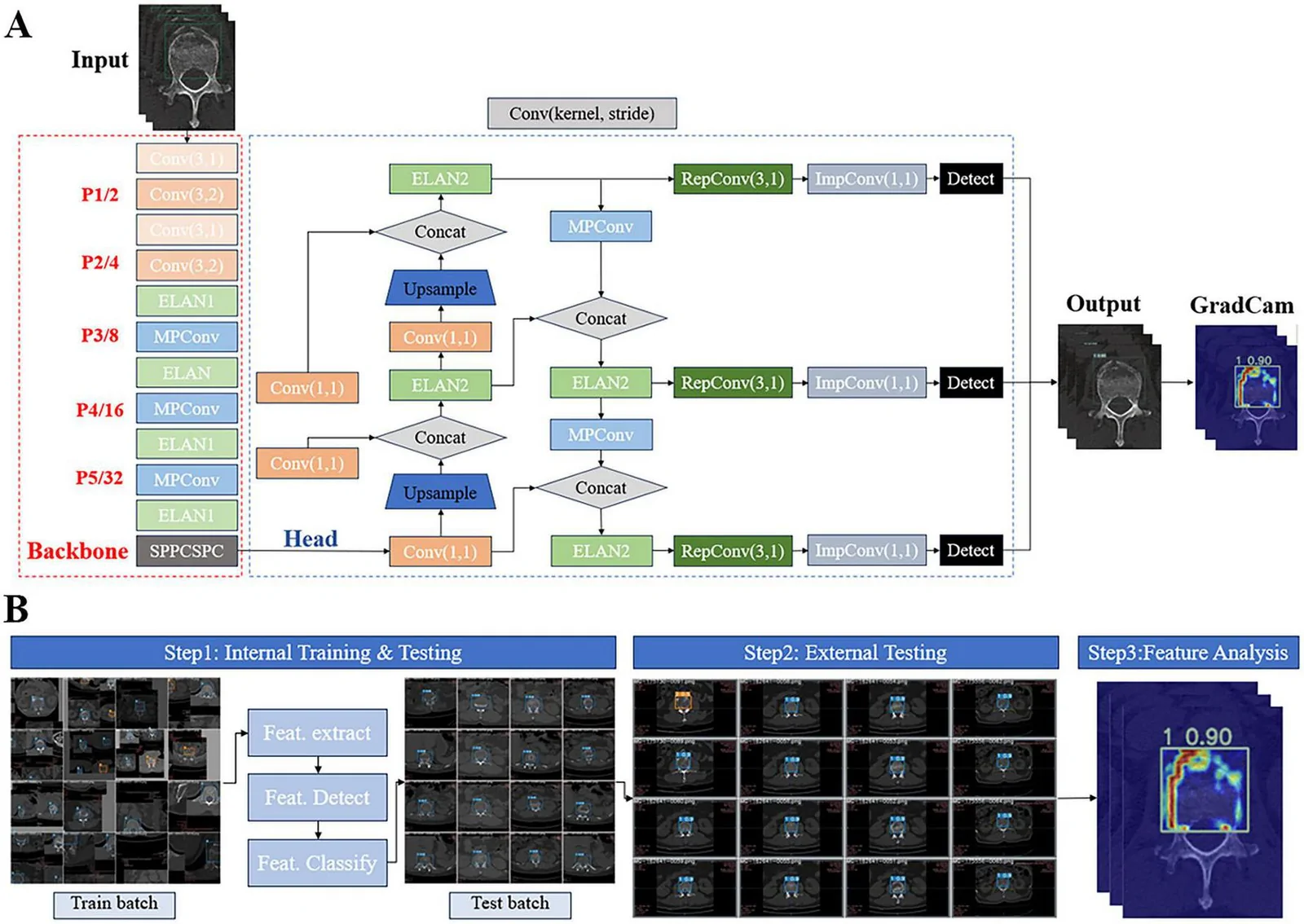
Deep learning (DL) model development. **(A)** The figure shows the complete model structure, from input to neural network model to output and the visualization analysis using Grad-Cam. **(B)** The Figure presents the core steps of the algorithm in this paper. Step 1 is internal training and testing (mainly including feature extraction, detection and classification), step 2 is external testing, and step 3 is feature visualization analysis.

The YOLO v7 model comprises four main components (Figure 2A): an input layer with data augmentation (color jitter, translation, scaling, fliplr, mosaic), a backbone network for feature extraction, a neck module employing ELAN (Efficient Layer Aggregation Network) and SPPCSPC (Spatial Pyramid Pooling with Cross-Stage Partial Connections) for multi-scale feature fusion, and a detection head that outputs class probabilities. For each fractured vertebra, all axial CT slices within the manually delineated rectangular ROI were resampled to 1024 × 1024 pixels (bilinear interpolation), HU values clipped to bone window (width 1500, level 300) and normalized to [0,1]. The backbone (modified CSPDarknet) extracts feature maps at three spatial scales (strides 8, 16, 32) to capture fine trabecular details and global vertebral morphology. Rather than using hand-crafted radiomic features, the model learns hierarchical representations directly from raw slices: early layers identify edge/texture primitives; intermediate layers form mid-level features such as “anterior cortical wavy discontinuity”, “focal trabecular clumping”, and “endplate depression angle”; deep layers encode holistic shape features including “vertebral body height reduction ratio” and “multi-slice consistency of cancellous attenuation”. The classification head uses two fully connected layers (256→*128*→2) with softmax. The model was adapted for vertebra-level binary classification: each axial slice outputs a two-dimensional vector (acute score, chronic score); the final vertebra-level prediction is the average acute score over all slices of that vertebra, thresholded at 0.5. Training used binary cross-entropy loss with positive class weighting (2.0) to address imbalance. Gradient-weighted Class Activation Mapping (Grad-CAM) applied to the final convolutional layer generates spatial heatmaps highlighting pixels most influential for the acute vs. chronic decision (Figure 2B).

### Comparison with radiologists

To compare the performance of the model with that of radiologists of different professional titles, three radiologists (Radiologist A is Zekai Zhu; Radiologist B is Yuequ Deng; Radiologist C is Chenyang Xing) were recruited to diagnose a randomly sampled subset of the internal and external testing set. The three radiologists were from Hospital 1 and comprised a junior radiologist with 5 years of experience, an intermediate radiologist with 10 years of experience, and a senior radiologist with 15 years of experience. The three radiologists evaluated a single, randomly selected axial CT image sequence of the fractured vertebrae from the test set without knowing the clinical information or the MRI results, to determine whether the vertebrae shown in that layer indicated an acute or chronic fracture. The diagnosis results of the 3 radiologists were collected and compared with those of the model. Blinding was used for both the model and the three radiologists.

### Statistical analysis

Data processing and analysis were performed via SPSS version 25.0 (IBM Corp., Armonk, NY, USA). For continuous clinical variables, independent samples *t*-tests were conducted between the two groups. Chi-square tests were employed to evaluate differences in categorical variables. Delong tests were performed to compare the diagnostic accuracy for acute and chronic OVCFs on CT between the DL model and the three radiologists. Bootstrapping was used to calculate 95% CIs. The model performance was evaluated in the internal testing set and external testing set respectively, including receiver operating characteristic (ROC) curve (AUC), accuracy, sensitivity, specificity, and F1 score. *P* < 0.05 was considered indicative of a statistically significant difference.

## 3 Results

### Baseline characteristics

The training set and the internal testing set were derived from Hospital 1. The training set included 8629 vertebral radiographs of 532 fractured vertebrae from 340 patients (mean age, 67.9 ± 13.7 years [SD]; 245 female and 95 male patients; 453 acute fractures and 79 chronic fractures). The internal testing set included 1570 vertebral radiographs of 92 fractured vertebrae from 60 patients (mean age, 68.1 ± 13.6 years [SD]; 43 female and 17 male patients; 74 acute fractures and 18 chronic fractures). The fractured vertebrae in the training set and the internal testing set were concentrated in the 12th thoracic vertebra and the 1st lumbar vertebra. The external testing set included 1158 vertebral radiographs of 86 fractured vertebrae from 70 patients (mean age, 64.9 ± 13.8 years [SD]; 54 female and 16 male patients; 60 acute fractures and 26 chronic fractures). The fractured vertebrae in the external testing set were concentrated in the 1st and 2nd lumbar vertebrae. The patient’s characteristics (age and sex) did not significantly differ between the training set and both testing data sets (Table 1).

**TABLE 1:** Patients’ characteristics and clinical information.

	Training set	Internal testing set	External testing set
Patients (*n*)	340	60	70
Vertebral radiographs (*n*)	8629	1570	1158
Age (y)	67.9 ± 13.7 (21–93)	68.1 ± 13.6 (21–93)	64.9 ± 13.8 (22–91)
Sex			
Female (*n*)	245	43	54
Male (*n*)	95	17	16
Acute (*n*)	453	74	60
Chronic (*n*)	79	18	26
Fractured vertebrae (n)			
T1	2	2	1
T2	1	0	0
T3	2	1	0
T4	2	1	0
T5	4	2	0
T6	2	1	0
T7	13	3	0
T8	7	2	0
T9	6	2	0
T10	7	3	0
T11	29	5	1
T12	108	18	11
L1	150	26	32
L2	93	9	17
L3	51	7	14
L4	42	6	8
L5	13	4	2

### Performance of the model and three radiologists

**FIGURE 3:**
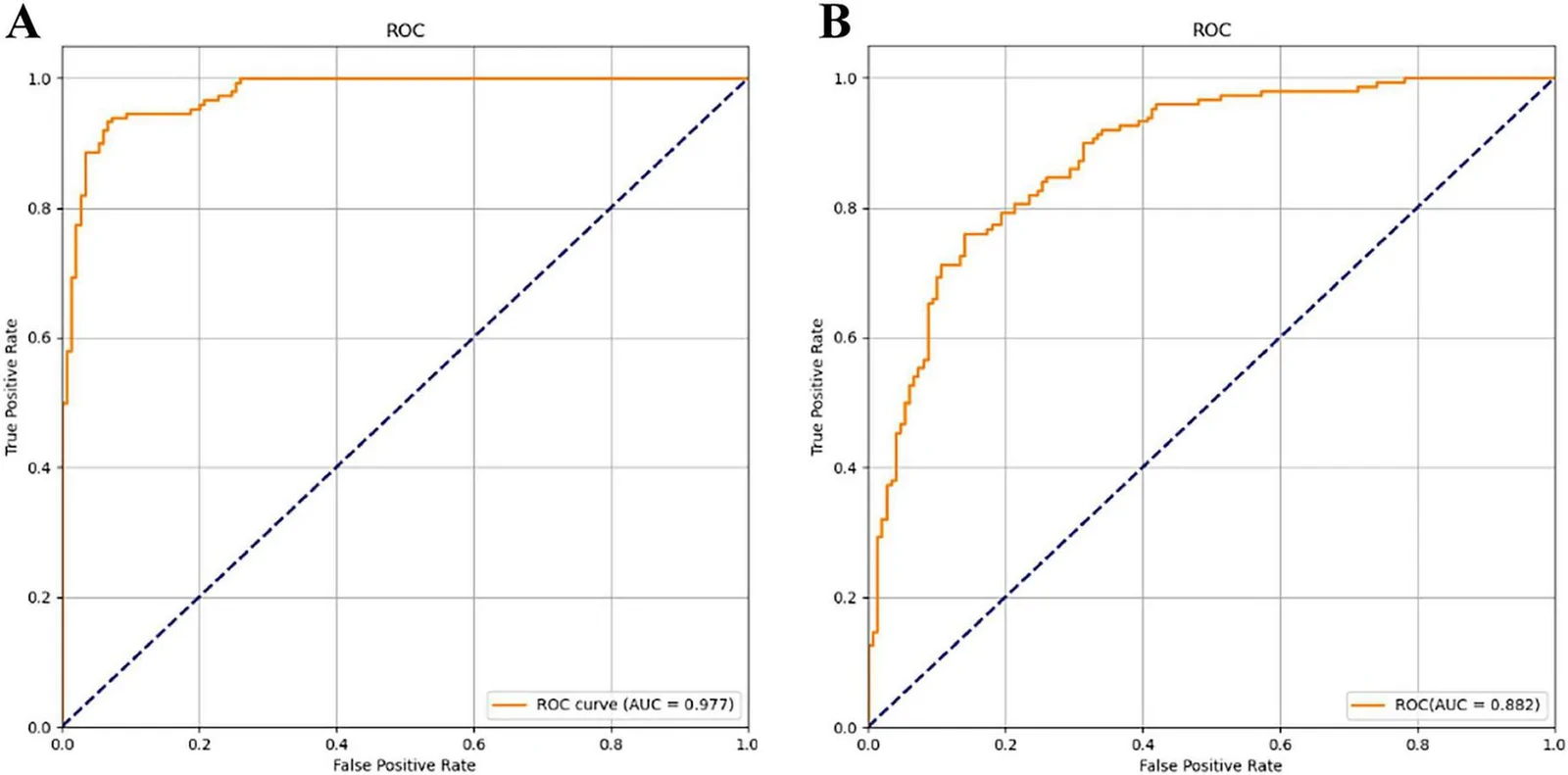
ROC curves. The ROC curves of the YOLO v7 model in the Internal and external testing set. **(A)** The ROC curve of the YOLO v7 model in the internal testing set. **(B)** The ROC curve of the YOLO v7 model in the external testing set.

The ROC curves for the model are shown in Figures 3A, B, and detailed performance metrics are summarized in Table 2. The inclusion of participants with chronic OVCF, those with suspected malignant VCFs, and those who underwent surgical treatments in the training process considerably improved model performance, resulting in higher AUC, accuracy, sensitivity, and specificity values in the internal and external testing sets. The model achieved an AUC of 0.937 (95% CI: 0.932, 0.991), an accuracy of 0.961 (95% CI: 0.930–0.977), sensitivity of 0.973 (95% CI: 0.958–0.994), specificity of 0.761 (95% CI: 0.728–0.784), precision of 0.936 ((95%CI: 0.920–0.966), and F1 score of 0.954 (95%CI: 0.913–0.975) in the internal testing set, and an AUC of 0.882 (95% CI: 0.839–0.894), accuracy of 0.852 (CI: 0.827–0.886), sensitivity of 0.895 (95% CI: 0.840–0.913), specificity of 0.780 (95% CI: 0.730–0.812), precision of 0.874 (95% CI: 0.839–0.892), and F1 score of 0.884 (95%CI: 0.846–0.903) in the external testing set. The performance of the three radiologists in the internal and external testing set are shown in Table 3. The best performing radiologist (A) achieved an accuracy of 0.789 (95% CI: 0.769–0.809), sensitivity of 0.817 (95% CI: 0.794–0.841), specificity of 0.737 (95% CI: 0.701–0.774), precision of 0.852 (95% CI: 0.829–0.874), and F1 score of 0.854 (95% CI: 0.838–0.870) in the internal testing set, and an accuracy of 0.837 (95% CI: 0.816–0.858), sensitivity of 0.833 (95% CI: 0.807–0.859), specificity of 0.846 (95% CI: 0.808–0.884) precision of 0.928 (95% CI: 0.909–0.946), and F1 score of 0.878 (95% CI: 0.855–0.901) in the external testing set. The performance of the YOLO v7 model is more excellent than that of radiologist A, especially in the internal testing set.

**TABLE 2:** Performance of the model.

Metric	Internal testing set	External testing set
AUC (95%CI)	0.937 (0.932–0.991)	0.882 (0.839–0.894)
Accuracy (95%CI)	0.961 (0.930–0.977)	0.852 (0.827–0.886)
Sensitivity (95%CI)	0.973 (0.958–0.994)	0.895 (0.840–0.913)
Specificity (95%CI)	0.761 (0.728–0.784)	0.780 (0.730–0.812)
Precision (95%CI)	0.936 (0.920–0.966)	0.874 (0.839–0.892)
F1 score (95%CI)	0.954 (0.913–0.975)	0.884 (0.846–0.903)

**TABLE 3:** Performance of three radiologists.

	Radiologist A	Radiologist B	Radiologist C
Internal testing set			
Accuracy (95%CI)	0.789 (0.769–0.809)	0.734 (0.712–0.756)	0.634 (0.610–0.658)
Sensitivity (95%CI)	0.817 (0.794–0.841)	0.764 (0.738–0.790)	0.672 (0.643–0.701)
Specificity (95%CI)	0.737 (0.701–0.774)	0.678 (0.639–0.717)	0.563 (0.522–0.605)
Precision (95%CI)	0.852 (0.829–0.874)	0.814 (0.789–0.839)	0.740 (0.711–0.768)
F1 score (95%CI)	0.854 (0.838–0.870)	0.788 (0.770–0.806)	0.704 (0.672–0.735)
External testing set			
Accuracy (95%CI)	0.837 (0.816–0.858)	0.773 (0.749–0.797)	0.687 (0.661–0.714)
Sensitivity (95%CI)	0.833 (0.807–0.859)	0.776 (0.748–0.805)	0.717 (0.686–0.748)
Specificity (95%CI)	0.846 (0.808–0.884)	0.765 (0.719–0.810)	0.616 (0.565–0.668)
Precision (95%CI)	0.928 (0.909–0.946)	0.886 (0.863–0.910)	0.816 (0.787–0.844)
F1 score (95%CI)	0.878 (0.855–0.901)	0.828 (0.798–0.857)	0.763 (0.742–0.785)

**FIGURE 4:**
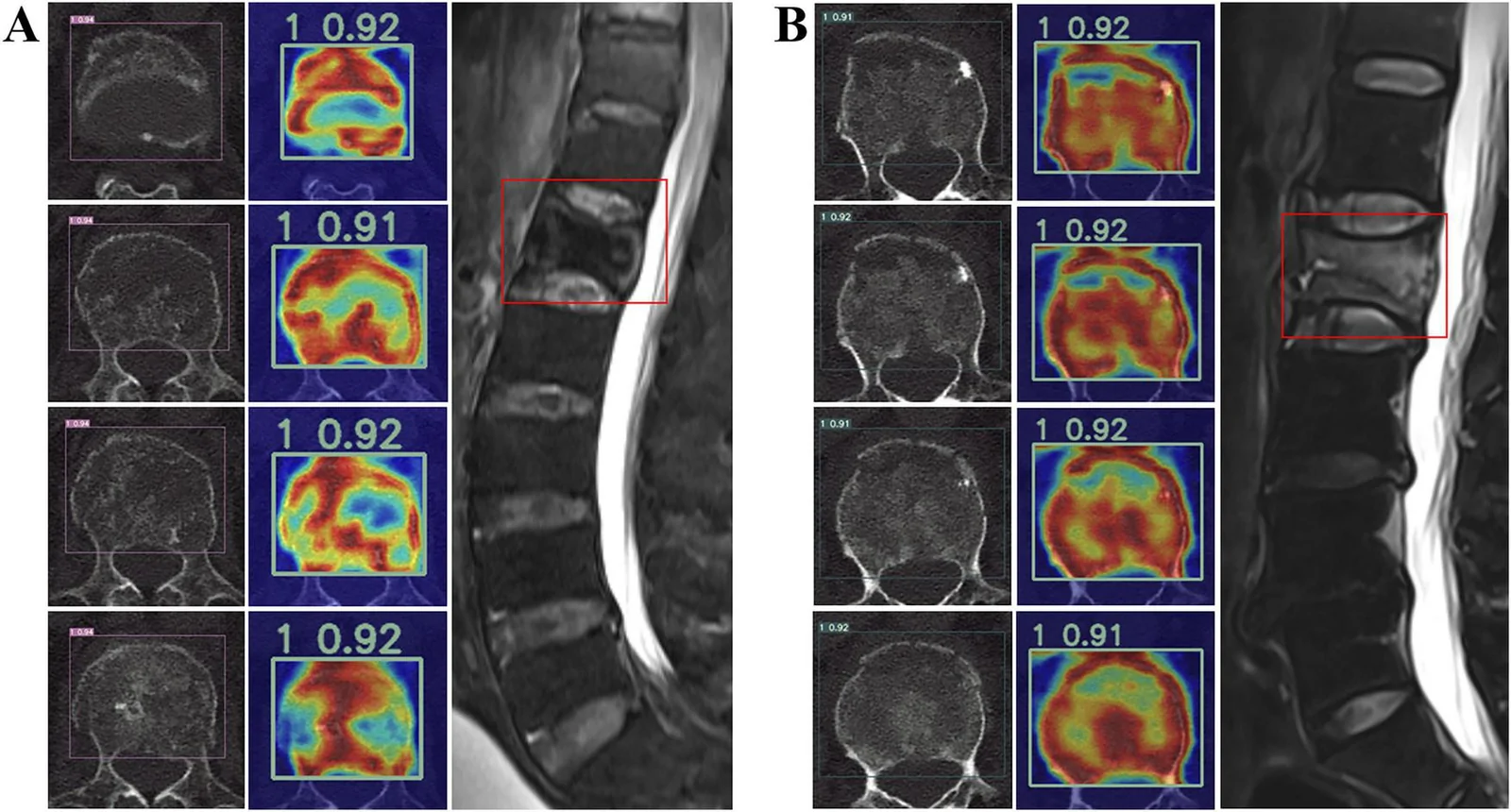
Visualizing CNN activations in images. Grad-CAM was used to localize areas used by CNN to identify OVCF within the images. The red rectangular box indicates the fractured segment. **(A)** Axial CT images and sagittal MRI images of chronic OVCF. **(B)** Axial CT images and sagittal MRI images of acute OVCF. Grad-CAM, gradient-weighted class activation mapping; CNN, convolutional neural network; OVCF, osteoporotic vertebral compression fracture.

Representative samples of chronic OVCF (Figure 4A) and acute OVCF (Figure 4B) in this study were selected. To evaluate the interpretability of the slice-level model, Gradient–weighted Class Activation Mapping (Grad-CAM) was employed, which identified salient pixels critical for distinguishing lesions from background tissue (23). This indicates that the model effectively focused on diagnostically relevant regions during prediction. When combined with slice-specific attention maps, the resulting segmentation output aids radiologists in locating, visualizing, and quantitatively assessing lesion areas.

## 4 Discussion

The differentiation of acute and chronic OVCFs remains a critical diagnostic challenge, with profound implications for clinical decision-making. In this study, we developed a deep learning (DL) model capable of distinguishing acute from chronic OVCFs using multidetector CT, achieving a diagnostic accuracy surpassing that of those of experienced radiologists while addressing key limitations of existing imaging modalities. An acute OVCF is characterized by bone marrow edema and trabecular microfractures and requires prompt intervention, such as vertebral augmentation, to reduce morbidity and mortality (24). Although MRI remains the gold standard for detecting edema, its contraindications (e.g., pacemakers, claustrophobia) exclude up to 26% of elderly patients (9), while SPECT is affected by limited availability and false negatives in early-stage fractures (25). Quantitative CT has demonstrated utility in detecting bone density changes associated with marrow edema (26). However, conventional CT windowing techniques lack sensitivity to subtle attenuation differences, underscoring the need for advanced computational approaches.

Recent advances in DL for OVCF detection highlight both progress and limitations. Liawrungrueang et al. (27) pioneered CT-based VCF detection using a deep artificial neural networks model (accuracy: 96.04%); however, their reliance on 2D analysis compromised generalizability. Liu et al. (28) achieved 83.77% accuracy and an AUC of 0.879 by improving automation via the MVITV2 model to differentiate early spinal tuberculosis from acute OVCF but failed to resolve trabecular patterns critical for acute-chronic differentiation. Lee et al. (29) achieved 87.84% accuracy with two-stage VCF detection with a segmentation and detection model but reported variability across scanner protocols. Similarly, Zhang et al. (30) employed deep learning radiomics for recurrent fracture prediction (AUC: 0.915); however, their reliance on data augmentation highlighted vulnerabilities to real-world heterogeneity.

Collectively, these studies reveal three unresolved challenges: First, existing approaches predominantly rely on sagittal reformations while neglecting transverse plane evaluations, resulting in insufficient characterization of trabecular destruction patterns. This dimensional oversight impedes comprehensive assessment of both morphological features and spatial distribution patterns of osseous microarchitecture deterioration. Second, the proliferation of diverse computational models demonstrates inconsistent performance stability when confronted with protocol variations in image acquisition parameters or processing pipelines. This architectural heterogeneity compromises generalizability across clinical imaging protocols, potentially undermining practical applicability. Third, several existing models exhibit critical deficiencies in external validation through multi-center trials or cross-protocol verification while simultaneously being limited by opaque decision-making processes. This dual limitation of unproven generalizability and poor model explainability creates significant barriers to clinical trust and suboptimal integration into diagnostic workflows.

Our model addresses these gaps through three innovations. First, the study employed axial full-sequence scans of target vertebrae as input features, in contrast to reconstructed sagittal plane projections, to achieve comprehensive characterization of vertebral anatomical integrity. Compared to conventional approaches that utilize sagittal maximum intensity projections for feature extraction, our methodology effectively mitigates the risk of overlooking critical pathological indicators such as cortical breaches and endplate fractures. Furthermore, when benchmarked against 3D volumetric reconstructions, our technique provided complete spatial coverage of fracture-affected vertebral bodies while maintaining computational efficiency. Quantitative evaluation demonstrated exceptional diagnostic performance, with the model achieving an area under the receiver operating characteristic curve (AUROC) of 0.937 (95% confidence interval [CI]: 0.932–0.991) on internal validation and 0.882 (95% CI: 0.832–0.894) on external testing. The system significantly outperformed the assessments of radiologists in both sensitivity and specificity. Through quantitative analysis of the complete volumetric data, the algorithm successfully resolved spatially heterogeneous trabecular injury patterns, thereby addressing the sampling bias inherent in current region-of-interest-based diagnostic protocols (30, 31). This multi-sequence analytical framework establishes a novel paradigm for detecting subtle osseous microarchitecture alterations associated with vertebral fractures.

Despite its strengths, this study has some limitations. First, the training data was derived from hospital-based CT-MRI pairs, and this may have led to an overestimation of the performance of the model in screening populations, where disease prevalence and imaging quality differ. Second, the study’s retrospective design may underestimate real-world radiologist performance, excluding low-quality scans and non-fractured vertebrae, limiting generalizability (32). Third, radiologists made diagnoses solely based on a single axial sequence, which severely limited their professional capabilities and might lead to a systematic underestimation of human performance. More clinically relevant validation should be conducted in a simulated real workstation environment, allowing radiologists to access complete image sequences and multi-planar reconstruction tools. Fourth, the model was trained and validated using MRI as the gold standard for differentiating acute from chronic OVCFs. However, the intended clinical use of the model is precisely in settings where MRI is unavailable or contraindicated. We did not independently validate the model’s performance against a true clinical outcome (e.g., treatment response or follow-up healing) in MRI-free scenarios. Therefore, generalization to populations without any MRI validation remains unproven. Fifth, the model’s training relied on manually delineated rectangular ROIs by radiologists. Although inter-rater variability was controlled through standardized annotation protocols, boundary discrepancies persisted among clinicians with varying experience levels when assessing vertebral compression severity. This dependency on manual annotation may hinder the model’s scalability in primary care settings. Sixth, although the model has achieved excellent performance in external validation, its performance has declined compared with the internal validation. Furthermore, the study’s sample size was relatively small and could have resulted in overfitting. In subsequent research, we intend to further increase the sample size. Therefore, we conclude that the DL model may serve as a second-reader aid for clinicians in settings where MRI is unavailable, provided that manual ROI delineation is feasible and that the model is applied to populations and scanner protocols similar to the training environment.

Future research should prioritize prospective multiethnic validation to refine predictive value calibration. Integrating multimodal data (e.g., DEXA-derived BMD and FRAX scores) via hybrid architectures could further enhance accuracy, while federated learning frameworks may mitigate scanner-specific heterogeneity. In our study, we developed a DL model that robustly differentiates acute and chronic OVCFs on CT, combining diagnostic precision with interpretable decision pathways. By addressing MRI’s accessibility barriers and CT’s diagnostic limitations, this tool shows promise for promoting equitable management of OVCF, particularly in underserved regions. However, because all enrolled patients were MRI-eligible, the model’s ability to replace MRI in MRI-ineligible or resource-limited settings remains unproven. Prospective validation in MRI-contraindicated populations against clinical outcomes is required before clinical deployment.

## 5 Conclusion

In this study, we developed a DL model to differentiate acute and chronic OVCFs on multidetector CT. The DL model performed well in the evaluation of both internal and external testing sets and outperformed imaging specialists. The DL model may help clinicians quickly identify the types of OVCFs and promote early personalized treatment strategies.

## Data Availability

The original contributions presented in the study are included in the article/supplementary material, further inquiries can be directed to the corresponding author.
